# Effects of the Structured Water Dance Intervention (SWAN) on muscular hypertonia in adults with profound intellectual and multiple disabilities

**DOI:** 10.1038/s41598-022-12716-1

**Published:** 2022-05-24

**Authors:** Lars-Olov Lundqvist, André Frank, Anna Duberg

**Affiliations:** University Health Care Research Center, Faculty of Medicine and Health, Örebro University, 70182 Örebro, Sweden

**Keywords:** Health care, Health services, Public health

## Abstract

This study aimed to evaluate the effect of Structured Water Dance Intervention (SWAN) on muscular hypertonia in individuals with profound intellectual and multiple disability (PIMD). Muscular hypertonia has a multitude of negative consequences for people with PIMD because it can lead to contractures, pain, mobility impairment, pressure ulcers that limits functional behavior as well as gross and fine motor function. Thirty-six individuals with PIMD in four Swedish regions were randomized to two groups in a multicenter, crossover design. Two withdrew participation, thus 34 individuals completed the intervention. The intervention was administered for 40 min once a week during a 12-week period. Outcomes related to muscular hypertonia were examined using the Modified Ashworth Scale (MAS), and based on accompanying assistants’ assessments. Hypertonia decreased from baseline to the end of the intervention period, as demonstrated by a decrease in MAS score. Hypertonia also decreased during the sessions, as shown by the assistants’ ratings. In conclusion, this study demonstrates that SWAN holds potential to reduce muscular hypertonia in people with PIMD and points out the importance of customized physical treatment alternatives. The study provides useful information for the design of future non-invasive, non-pharmacological interventions to reduce muscular hypertonia in PIMD.

## Introduction

Adults with profound intellectual and multiple disabilities (PIMD) have a combination of intellectual disability and physical impairments. They are constantly dependent on others, and have extensive need for assistance with activities of daily living^1^. A major issue affecting the everyday life of people with PIMD, who are commonly diagnosed with cerebral palsy, is the presence of motor disorders mainly due to muscular hypertonia, which is manifested as an increase in resistance to passive stretch. Spasticity, as a component of hypertonia, can be described as a disturbance in sensorimotor control, resulting from an upper motor neuron lesion, presenting as intermittent or sustained involuntary activation of muscles^2^. Muscular hypertonia has negative consequences because it changes the viscoelastic properties of soft tissues and joints, which can lead to contractures, pain, mobility impairment, and development of pressure ulcers^3^ resulting in limitations in gross motor and fine motor skills. Hypertonia can furthermore negatively affect quality of life by impairing activities of daily living and care delivery^4^. With a prevalence of 76%, hypertonia is very common among people with PIMD^5^.

Given the prevalence and potential negative consequences of hypertonia among individuals with PIMD, several treatments have been developed to mitigate its negative effects. Perhaps the most common medical treatment is pharmacological (e.g., baclofen, or injection of botulinum toxin), or orthopedic surgery^6^. Some medical treatments may be easy to use, but they have unwanted systemic effects, which can outweigh the potential benefits they may provide^7^. Consequently, a number of non-invasive and non-pharmacological treatments with fewer side effects have been developed, including physical therapy, occupational therapy, and hydrotherapy^8,9^. The effect of these treatments is less known due to the lack of high-quality evidence studies^9–11^. Notwithstanding, hydrotherapy is a potentially effective treatment for people with PIMD because of the unique characteristics of the water such as the buoyancy, the resistance and the temperature^12^. That is, hydrotherapy can offer opportunities of performing movements not possible on land, and the warm water may offer an enriched sensory experience^13^. Given these advantages, hydrotherapy has shown beneficial effects on joint mobility, physical function, pain, and quality of life in adults with musculoskeletal conditions^14,15^ as well as in children with cerebral palsy although they have less severe motor impairment^16,17^. Based on the positive effects of hydrotherapy and that dance in general is a well-adapted and feasible activity that can improve psychosocial aspects and motor skills in people with neurological disorders^18^, the Structured Water Dance Intervention (SWAN) was developed to improve aspects of health, including muscular hypertonia, in adults with PIMD and to give them opportunities for movement enjoyment and social interaction. Structured water dance is an aquatic group activity in which the participant dances with a support person in a warm water pool.

However, besides a pilot study^19^ indicating beneficial effects, there has been no controlled evaluation. Therefore, the aim of this study was to evaluate the effect of SWAN on muscular hypertonia in individuals with PIMD. More specifically, we hypothesized that SWAN has a beneficial effect on hypertonia, producing a decrease in response to a single water dance session, and also resulting in a reduction in hypertonia after the whole intervention period compared to before the intervention.

## Materials and methods

### Study design

**Figure 1:**
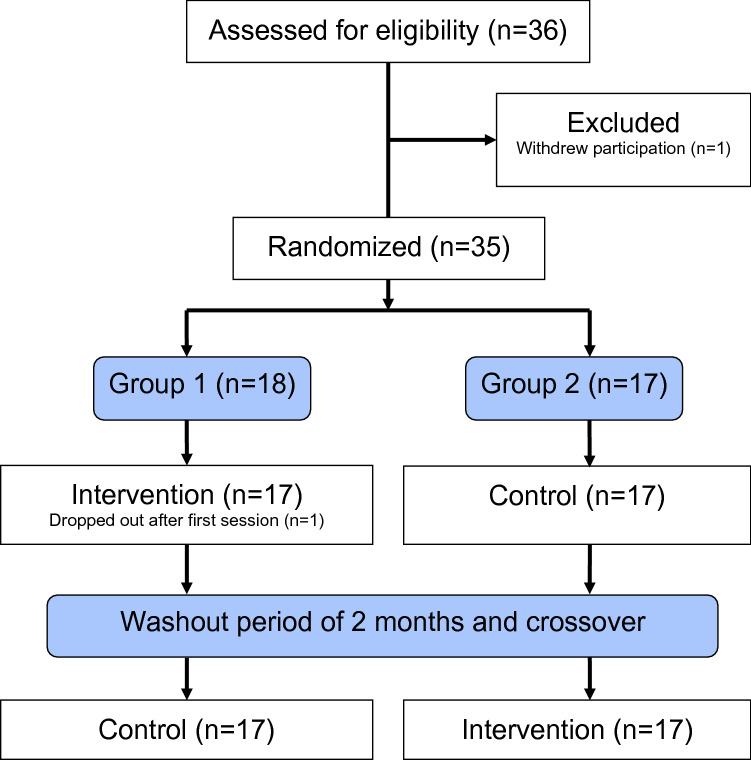
The crossover study design.

The present study was part of a multicenter randomized intervention study enrolling participants from adult habilitation centers in four Swedish regions. Details of the study design have previously been reported^20^. Each center had two groups: an early intervention group (Group 1) and a late intervention group (Group 2), see Fig. 1. The participants were randomized to Group 1 or Group 2 by minimization^21^ based on gender, age, and hypertonia at baseline, using the Minim software^22^. Group 1 received SWAN when they entered into the study while Group 2 acted as a control group. After a washout period of 2 months, Group 2 received the intervention, while Group 1 returned to their normal activities. Thus, the participants were expected to complete both intervention and control conditions.

### Participants

Participants were recruited by local physiotherapists in collaboration with the research team. Criteria for inclusion were being aged 18–65 years, being on the PIMD spectrum with a motor impairment corresponding to Gross Motor Function Classification System (GMFCS) level IV or V^23^ and with profound intellectual disability according to the International Classification of Diseases, tenth revision (ICD-10), of the World Health Organization (WHO)^24^. In addition, participants should be accustomed to water and not find discomfort of activities in water. Criteria for exclusion were severe hearing impairment or deafness (as music was played to enhance the aquatic activity), and infections or ulcers (due to the risk of infection through pool water). Based on available muscular hypertonia data from the five persons who participated in the pilot study^19^, a pre-power analysis using an online service (www.stat.ubc.ca) indicated that 40 participants were needed to achieve a study power of 80% and an alpha of 5%. However, we managed to recruit only 36 individuals (20 men and 16 women) and unfortunately, two of the women withdrew their participation at the beginning of the study. One withdrew participation before the first session, and the other attended the first SWAN session but withdrew further participation due to stomach problems and issues with tolerating group activities. Therefore, 34 individuals (20 men and 14 women) completed the intervention and the control conditions. A description of the study population based on caregivers’ information and the medical records is presented in Table 1.

**Table 1:** Characteristics of the study participants at baseline.

Variable	
Age, yrs, M (range)	33.8 (21–53)
Gender, n (%)	
Men	20 (59%)
Women	14 (41%)
Diagnosis *, n (%) #	
Epilepsy (G40)	29 (85%)
Cerebral palsy (G80.9)	24 (71%)
Other brain disorders (G93)	3 (9%)
Autism spectrum disorders (F84)	3 (9%)
Rare diseases (e.g., Rett, Kabuki syndrome)	3 (9%)
Asthma/allergy (J45)	3 (9%)
Visual impairment (H54)	2 (6%)
Comorbidity †	30 (88%)
Motor disorder, n (%)/M (SD)	
Spasticity (MAS > 0)	34 (100%)
Mild increased resistance (0 < MAS < 2)	9 (26%)
Moderately increased resistance (MAS ≥ 2)	12 (35%)
Severely increased resistance (MAS ≥ 3)	9 (26%)
Profoundly increased resistance (MAS ≥ 4)	4 (12%)
Average MAS score (0–5)	2.48 (0.98)
Limited mobility (1–5)	4.03 (1.14)
Spasticity-related medical treatment (e.g., Baclofen, Stesolid, Buccolam)	22 (65%)
Epilepsy-related medical treatment (e.g., Lamictal (lamotrigine), Keppra, Tegretol)	28 (82%)

*M* mean, *MAS* Modified Ashworth Scale, *SD* standard deviation.

*ICD-10 code in parenthesis.

^#^In addition to intellectual disability. Since some patients had more than one diagnosis, percentages will not sum up to 100%.

^†^Two or more diagnoses in addition to intellectual disability.

### The Structured Water Dance Intervention

Structured water dance was performed as a group activity in groups of four to five participants in a warm pool 33–35 degrees Celsius (91–93 degrees Fahrenheit), held once a week for 12 weeks. The sessions followed a structured 40-min program including a fixed playlist of nine music tracks. The tracks were chosen to stimulate the rhythm of the movements performed, to support relaxation, and to elicit different emotions.

The dance sequences included in the SWAN session focus on adapted passive movements of the participant’s body, enjoyment, interaction and on stimulation of the senses. The participant is lying in the supine position during the session except for two dances where he or she is held in an upright position, sitting or standing. Passive movements of different body parts of the participant is performed throughout the session by the assistant. For example, during one of the music tracks dancing is performed by moving the participant’s arms, and during another music track dancing is performed by swinging together in an upright position. Two assistants accompany each participant. One of them is in the pool and functions as a dance partner and the other is at the poolside and assists when the participant enters or leaves the pool, as well as helping out with floating devices and other practical matters. The sessions are led by two instructors, usually a physiotherapist and a physiotherapy assistant. One of them guides the group from the poolside, and the other is in the water to assist the participants and their support persons.

### Outcome measures

**Figure 2:**
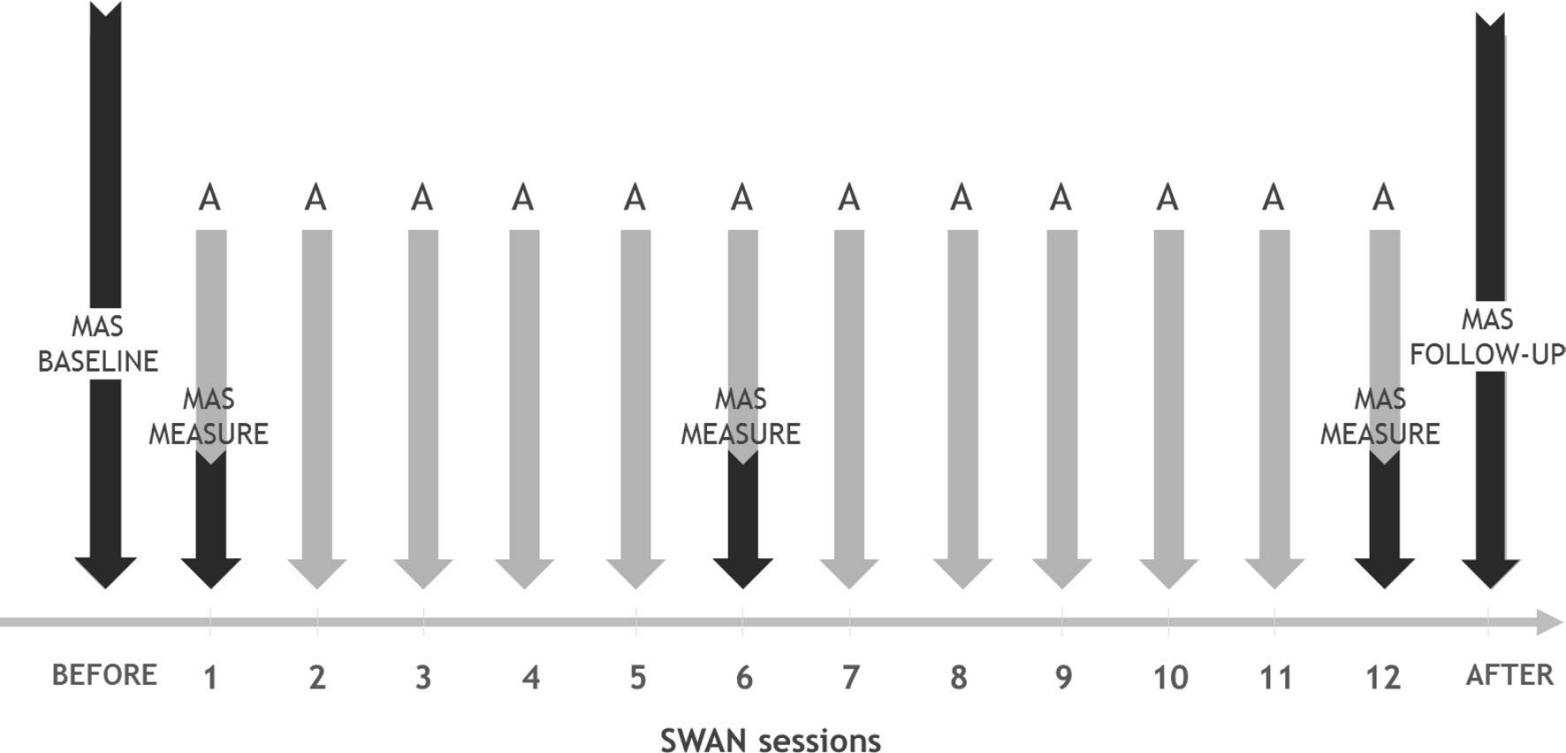
Outcome measure timeline. *A* Assistants’ assessment before, during, and after each session; *MAS* Modified Ashworth Scale assessment.

We used two measures to quantify change related to muscular hypertonia: the Modified Ashworth Scale (MAS) and assistant assessments. For the timeline of the study, see Fig. 2.

#### The Modified Ashworth Scale

The MAS^25^ assesses the muscular resistance occurring during a quick passive movement. The response is scored on a 6-point scale, consisting of grades 0, 1, 1+, 2, 3, and 4, where 0 = no increased resistance in the muscles, and 4 = muscles being rigid during flexion or extension. In this study, the MAS grades were transformed into 0, 1, 2, 3, 4, and 5 scores. The MAS has been shown to be reliable when used to assess muscle tone in persons with PIMD^26^.

The MAS was applied before and after the intervention period, and in conjunction with the first, sixth, and twelfth SWAN session. One of the authors (A.F.), a physiotherapist with more than 20 years’ experience of working in disability, performed the MAS assessments except in four cases where the participants did not come to the appointed pre-intervention assessment. These participants were instead assessed by the local physiotherapist.

The pre- and post-intervention assessments were conducted at the habilitation centers, and in two cases at the participant’s home. The session assessments were performed right before transfer into the pool, with the participant lying on a shower bed in the supine position or sitting (two participants) in a shower commode chair, and immediately after the session before the participant left the pool. The flexor muscles of the right and left elbow and the extensor muscles of the right and left knee were assessed. The assumption was that the whole body was involved in the aquatic activity, so the mean MAS score based on the four measures was used.

#### Assistant assessments

The two assistants together assessed the participant’s degree of muscular hypertonia on a 5-point Likert scale, from 0 = no hypertonia at all, to 4 = very much. The assessment was completed three times in conjunction with each session: before the session started when the participant was in the locker room, during the water dance session, and finally after the session when the participant was back in the locker room. Before measurement the assistants were briefed on the assessment form by the instructors, who before the study underwent a one-day training in the theoretical basis, design, performance and outcome measures of the SWAN project.

### Data analysis

Linear mixed model analysis with restricted maximum likelihood (REML) was chosen as analysis method because it uses all the available information in data in a repeated-measures design and is robust in handling missing data^27^. A separate linear mixed model was built for each outcome variable. Unstructured components analysis was used to account for within-subject correlation over time. Since there were only two dropouts, no specific dropout analysis was conducted. All analyses were conducted using the IBM Statistical Package for the Social Sciences for Windows, version 25.0 (IBM Corporation, Armonk, NY, USA). The level of statistical significance was set at *p* ≤ 0.05.

### Ethical considerations

The study was performed in accordance with the Declaration of Helsinki^28^. In view of the profound disability of the participants, the participants’ legal guardians gave informed consent after reading written information about the study and further receiving information from the researchers via telephone or in person. Although the risk of discomfort or harms in connection with the intervention is not considered higher than in a regular course of treatment, harms related information was collected by instructing the participants assistants and legal guardian to be vigilant on signals of discomfort or harms and to immediately notify the SWAN leader or the responsible researcher if observed.

### Ethics approval and consent to participate

The study was approved by the Regional Ethical Review Board in Uppsala, Sweden (approval number: 2018/070) and was carried out in accordance with the Declaration of Helsinki. Written informed consent has been obtained from the legal guardians of all participants.

## Results

### Baseline characteristics

Characteristics of the study group are given in Table 1. There were more men (n = 20) than women (n = 14) participating in the study. All participants had some level of hypertonia, with 74% scoring 2 or higher on the MAS. All of them had limited mobility, according to the caregivers who reported that the limited mobility complicated the participants’ care. Epilepsy and cerebral palsy were the most prevalent diagnoses. Approximately two thirds of the participants were reported to have hypertonia-related medical treatments, predominantly baclofen. Two participants received botulinum toxin treatment shortly before or during the treatment period.

### Adherence to the intervention

The participants attended between three and twelve sessions, with a mean attendance of 9.3 sessions and a median attendance of ten sessions. Twenty participants (59%) attended ten or more sessions. Only two participants (6%) attended fewer than six sessions. The reason for their low attendance was arising health issues not related to the intervention. No harms were reported by the assistants or legal guardians.

### The Modified Ashworth Scale

#### Effects on muscular hypertonia—pre-post-intervention period

**Figure 3:**
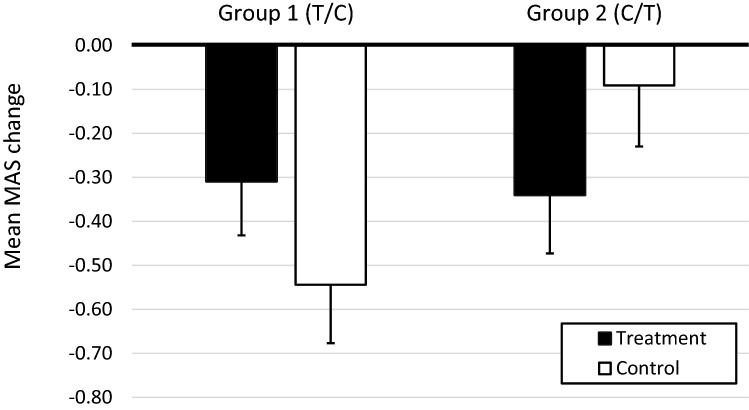
Mean change in Modified Ashworth Scale (MAS) score from baseline to post-treatment intervention, including also the control period, for Group 1 (treatment first/control period second) and Group 2 (control condition first/treatment second). Error bars represent 1 standard error.

The linear mixed model analysis showed no main effect of group or treatment; however, a significant group by treatment interaction effect was seen (*F*_(1,32)_ = 10.50, *p* = 0.003). As presented in Fig. 3, MAS scores in Group 1 decreased from baseline to post-treatment (*F*_(1,16)_ = 4.77, *p* = 0.044). However, the MAS scores continued to decrease during the subsequent control period compared to baseline (*F*_(1,16)_ = 10.38, *p* = 0.006). In Group 2, which started in the control condition, there was no significant decrease in MAS between baseline and the control condition (*F*_(1,16)_ = 0.78, *p* = 0.389). There was, however, a significant decrease in MAS between baseline and the post-treatment period (*F*_(1,16)_ = 5.77, *p* = 0.029).

#### Effects on muscular hypertonia—pre-post-sessions

**Figure 4:**
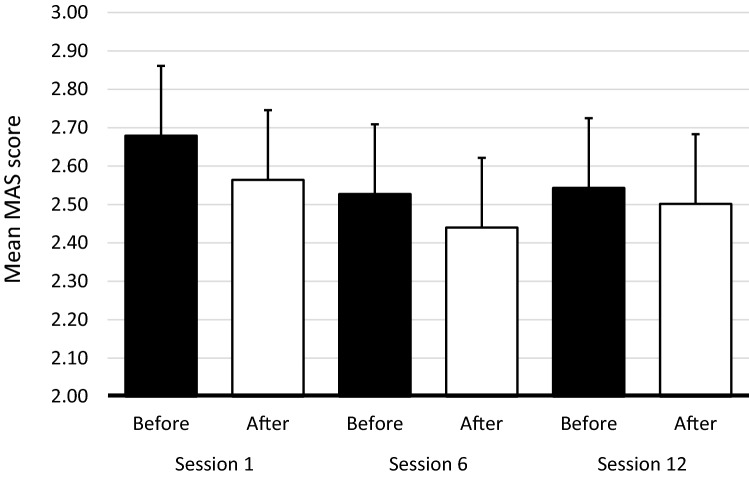
Mean Modified Ashworth Scale (MAS) score before and after sessions 1, 6, and 12. Error bars represent 1 standard error.

The MAS score was assessed directly before and directly after sessions 1, 6 and 12. As shown in Fig. 4, MAS scores decreased within each session. The decrease was, however, small and the linear mixed model analysis showed no significant decrease in MAS scores within or across sessions.

### Assistant assessments

**Figure 5:**
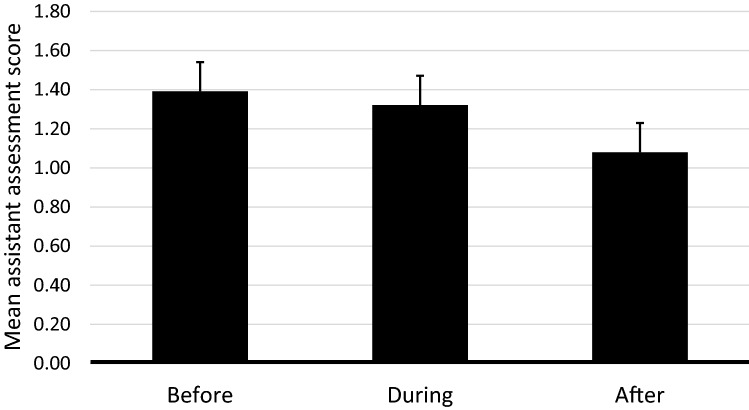
Mean score of assistants’ assessments of participants’ degree of hypertonia before, during, and after the twelve sessions of Structured Water Dance Intervention (SWAN). Error bars represent 1 standard error.

The two accompanying assistants together assessed the participant’s hypertonia before, during, and after each of the twelve sessions. The assistants’ ratings indicated a reduction in hypertonia across the twelve sessions which only approached significance according to the linear mixed model analysis (*p* = 0.061). However, when analyzing the average ratings of all twelve sessions, a significant change in participants’ hypertonia within sessions was revealed (*F*_(2,502)_ = 6.47, *p* = 0.002). As shown in Fig. 5, there was a significant decrease in hypertonia between the ratings made directly before and directly after the sessions (*F*_(1,330)_ = 11.20, *p* < 0.001). There was also a significant decrease between the ratings during and after the sessions (*F*_(1,325)_ = 6.93, *p* = 0.009), but there was no significant decrease in hypertonia between the ratings before and during the sessions.

## Discussion

The aim of this study was to evaluate the effect of SWAN on muscular hypertonia in individuals with PIMD. The results showed that SWAN significantly decreased muscular hypertonia before and after water dance sessions (measured by assistant assessments and using tendency in MAS) and pre- and post-intervention when comparing the two groups (MAS only). However, in Group 1, MAS scores continued to decline during the subsequent control condition, which could lead to questioning the integrity of the crossover design. This continued decline may indicate that there is a potential long-lasting effect of the intervention. Future evaluations could address this issue by using multiple follow-ups.

The average decrease in MAS score, post-treatment, of − 0.33 is at the low end with regard to a minimal clinically important difference (MCID), according to Chen et al.^29^. This indicates a small effect size of SWAN, although it should be noted that the evaluation of MCID of the MAS, by Chen et al.^29^, was based on stroke patients, who have CNS lesions that are mostly acquired later in life while the lesions or other injuries of the CNS of the participants of the present study were acquired early in life, thus making comparisons more difficult. Therefore, it is difficult to determine whether the observed decrease in hypertonia in our study is on par with what can be expected in adults with long-term, chronic hypertonia. However, results of a twenty-week power-assisted exercise intervention for adults with profound intellectual and multiple disabilities reported a non-significant change in MAS of 0.06 between baseline and follow up^30^. Thus, a change of − 0.33 in MAS following the SWAN intervention may be better than expected. Nevertheless, research addressing the question of MCID of the MAS in PIMD is warranted in evaluation of clinical interventions in this group of patients. Compared to pharmacological methods, such as botulinum toxin, the decrease in MAS scores in our study is similar, but at the lower end of results for children with cerebral palsy in studies^31,32^ reporting an average decrease of between − 0.40 and − 1.00 in MAS scores for upper and lower limbs at 1–2 months’ post-treatment.

Few studies have evaluated hydrotherapy for people with PIMD. However, in line with our results, a study on individuals with severe to profound developmental disabilities participating in an aquatic physical therapy program showed decrease in muscle tone (using the MAS) and improvement in additional measures, such as passive range of motion and caregivers’ evaluations of burden of care^33^. Another study investigating the suitability of an aquatic program for people with severe and profound intellectual disabilities by interviewing staff, found that aquatic intervention was perceived to provide freedom of movement, social interaction, and wellbeing^34^, supporting our results showing that aquatics can be beneficial for this group. Similar positive results on freedom of movement, less stiffness and wellbeing were found from interviews with adults with neurological movement disorders who had participated in hydrotherapy^35^. Additionally, a study on children with cerebral palsy showed that children at all GMFCS levels improved in MAS scores after participating in an aquatic exercise group^16^. Therefore, our results are in line with previous research.

To put the present results in relation to pharmacological methods, we can conclude that non-pharmacological methods, such as SWAN, can be beneficial and can serve as a complement to pharmacological treatments for individuals with PIMD and potentially for patients with other neurological conditions suffering from hypertonia^36^. Therefore, it is recommended that health care providers find solutions for the delivery of these treatments, especially since individuals with PIMD have difficulty in describing their needs and asserting their rights themselves. Although the participants were adults with a long history of hypertonia and although the effects of SWAN were relatively small, it is noteworthy that treatment can reduce hypertonia in adults even after long periods without treatment^4^. Nevertheless, maintaining function as rehabilitation goal is just as relevant as improving functions.

Before concluding, there are some study limitations to consider. Firstly, the condition of PIMD is relatively rare and our power analysis prior to the start of the study indicated a required minimum sample size of 40. Despite using a multicenter design, we only managed to recruit 36 participants. Therefore, we can conclude that the sample size was somewhat too small according to the power calculation, which increases the risk of missing actual differences, i.e., making type II errors.

Secondly, the MAS has never been systematically tested in water. Since water may cause an additional resistance that distorts the assessments, we piloted this prior to the study and found that the MAS performed satisfactorily. The session assessments of MAS scores were performed right before the participants were transferred into the pool and again immediately after the session before they were moved out of the pool. The alternative procedure of assessing MAS scores at the poolside, post-session, was not followed as it likely would have increased muscle tone from participants being cold.

Thirdly, when assessing hypertonia using MAS in individuals with PIMD, the cognitive disability may cause the participant to experience stress since he or she may be unable to understand the purpose of the assessment and the instructions given by the physiotherapist from the research team^2^. In our study, in consideration of this, the assessing physiotherapist prepared the participant by talking calmly and by letting the familiar staff member or parent assist him in making the test situation as relaxed and safe as possible. Using assistants’ assessment of hypertonia provide other challenges. The assessments were made mainly through the assistant’s physical contact with the participant during the water dance in the pool and the handling in connection with the dressing and shower procedures before and after the sessions. Although being less structured and systematic than MAS, the assistants’ assessments provide a general measure of hypertonia by someone who is familiar with the participant and therefore supplement the MAS assessment.

Fourthly, adults with PIMD who have had lifelong hypertonia and spasticity commonly have contractures in muscles and joints, which in some cases made the assessments difficult to perform. In the study by Van Timmeren et al.^5^, contractures were found in 32% of the population observed. In fact, already in children with CP, fibrotic changes in spastic muscles can be seen and may be related to muscle stiffness and possibly to the development of contractures^37^. Despite the potential for an adverse impact of chronic hypertonia on the MAS assessment, we consider the MAS among the most adequate measures to use in these circumstances.

Fifthly, as with many non-pharmacological treatments, blinded assessments were impossible to perform as the caregivers and the assistants knew when the participant was receiving the intervention and when not. However, we used assessment triangulation with independent assessors on the same construct, with the intention to reduce this risk of assessment bias.

Finally, the evaluation of SWAN was made on the intervention as a whole. Therefore, it is not possible to determine the significance of the individual elements of the intervention. The elements of warm water, music, social interaction, and participation in an organized activity that breaks everyday routines are all important and may all have affected the outcome. Given that several of the participants had regular aquatic activities even as part of the control group as we wanted them to continue with their normal activities, the effect of SWAN may be greater than observed. In consideration of these limitations, the present study provides support for a positive effect of SWAN on hypertonia in individuals with PIMD and encourages future studies that can address the weaknesses of the present study.

The present study has a number of clinical implications. There are few evidence-based, non-invasive non-pharmacological methods to address the needs of people with PIMD. The present study aimed to evaluate one possible effect of SWAN, namely, its effects on muscular hypertonia. Compared to regular physical activity in water, dance and music add certain characteristics to the activity, such as engagement and enjoyment, which can stimulate motivation and commitment to continue participation in interventions over time^38,39^. Since SWAN as well as other aquatic interventions e.g., Adar et al.^16^ may have beneficial effects in addition to reduced muscular hypertonia, clinicians who might consider using SWAN should base their decisions not solely on the need for reduced hypertonia, but on a holistic view of the needs of persons with PIMD. This study did not compare SWAN with other interventions addressing hypertonia in PIMD, thus we do not know whether it is more or less efficient than other non-pharmacological interventions based on hydrotherapy, physical therapy, or occupational therapy. However, we believe that an ongoing intervention that is perceived as beneficial should not be replaced in favor of SWAN.

This is the first attempt to evaluate the effect of SWAN in reducing muscular hypertonia. As illustrated above and recently thoroughly elucidated by Bea Maes et al.^40^, evaluating any method on groups of people with rare and severe disorders is a challenge. Therefore, the present results need to be replicated in another and preferably larger sample as well as in other settings and contexts. Future studies may also focus on more clearly determining the constituent ingredients of SWAN, such as the effect of the music, the movements in warm water, the social interaction, the performance in a group versus individually, and, in comparison to other treatments, possible synergy effects with conventional pharmacological treatments, as suggested by Naro et al.^8^. We found short-term effects on muscular hypertonia. However, longitudinal studies are needed to reveal long-term effects of SWAN.

In conclusion, the present study demonstrates that SWAN is a non-invasive, non-pharmacological intervention with potential to reduce muscular hypertonia in people with PIMD. The study points out the importance of customized physical treatment alternatives for the target group and may provide useful information when designing future interventions on reducing muscular hypertonia.

## Data Availability

The datasets generated during the current study are not publicly available due to legal and ethical restraints. Study data are available from the corresponding author on reasonable request.
